# ALZHEIMER’S DISEASE AND OPPORTUNITY FOR POLICY CHANGE

**DOI:** 10.1093/geroni/igad104.1237

**Published:** 2023-12-21

**Authors:** Bailee Brekke, Christina Mu, Alisha Thompson, Patricia D’Antonio

**Affiliations:** Miami University, Oxford, Ohio, United States; University of South Florida, Tampa, Florida, United States; LSU, Baton Rouge, Louisiana, United States; The Gerontological Society of America, Washington, District of Columbia, United States

## Abstract

Amidst the introduction of breakthroughs to slow the progression of Alzheimer’s Disease, hesitation remains among people living with dementia and their care partners. Currently, over 7 million Americans are impacted by Alzheimer’s Disease and another 11 million Americans provide unpaid care for someone living with the disease. Despite the immense efforts made to develop treatments for those diagnosed, barriers in accessibility remain. As a GSA policy intern, I attended multiple coalition meetings centered around advocating for equitable access to new treatment options. I also analyzed legislation aimed at improving early detection, delaying disease progression, and improving access to treatments for people with Alzheimer’s disease and related dementias (ADRD). While following the work of agencies including, the Centers for Medicare and Medicaid Services (CMS), I found that noteworthy progress has been made in terms of coverage expansion, but GSA and other organizations will need to continue to advocate for those older adults who have been left out of the equation. It is important to recognize the considerable progress that has been made and the immense efforts in support of people affected by this disease and their care partners; however, it is evident that Alzheimer’s Disease must remain at the forefront of bipartisan policy initiatives to ensure that legislation is passed, and these barriers are alleviated.

